# Lessons Learned From the Function and Behavior Focused Care Trial: From Efficacy to Implementation

**DOI:** 10.1093/geroni/igaa057.2806

**Published:** 2020-12-16

**Authors:** Elizabeth Galik, Marie Boltz

## Abstract

Long term care residents with dementia are sedentary, experience rapid functional decline, and frequently exhibit behavioral and psychological symptoms of dementia. Our prior cluster, randomized controlled trial among 336 residents with moderate to severe dementia in 12 nursing homes demonstrated that it is possible to increase time spent in physical activity and decrease resistiveness to care through a theory based intervention, Function and Behavior Focused Care (FBFC). FBFC is based on the Social Ecological Model and Social Cognitive Theory and focuses on having long term care staff cue, model, and assist residents with dementia to engage in physical activity and perform functional tasks. Learning from prior work, it was noted that future implementation of FBFC would benefit from de-implementing inaccurate care practices, such as restricting resident mobility and providing custodial care and also by engaging a full stakeholder team in intervention activities. Additionally, there were measurement issues, such as the use of actigraphy with a sedentary, cognitively impaired population, and the need to assess the quality of care interactions between residents and staff. This symposium will review lessons learned from the FBFC trial and will discuss 1) facilitators and barriers to the implementation of the FBFC intervention within long term care settings; 2) measurement opportunities and challenges with a cognitively impaired long term care population; and 3) adaptation of the FBFC intervention to be appropriate for a dissemination and implementation trial that incorporates the Synthesis Model of De-Adoption and the Evidence Integration Triangle implementation strategy.

